# Ependymal cells SCOre sweet cerebrospinal fluid

**DOI:** 10.1371/journal.pbio.3002323

**Published:** 2023-09-22

**Authors:** Luke L. Liu, Ryann M. Fame

**Affiliations:** Department of Neurosurgery, Stanford University, Stanford, CA, United States of America

## Abstract

The subcommissural organ (SCO) is a secretory tissue located on the roof of brain’s third ventricle. This Primer explores a new PLOS Biology study revealing that the SCO responds to glucose by secreting signaling molecules into the cerebrospinal fluid (CSF), thereby decreasing the local ependyma-driven CSF movement.

## Teaser

The subcommissural organ (SCO) is a secretory tissue located on the roof of the brain’s third ventricle. A new study published in *PLOS Biology* finds that the SCO responds to glucose by secreting signaling molecules into the cerebrospinal fluid (CSF), thereby decreasing the local ependyma-driven CSF movement.

**Fig 1:**
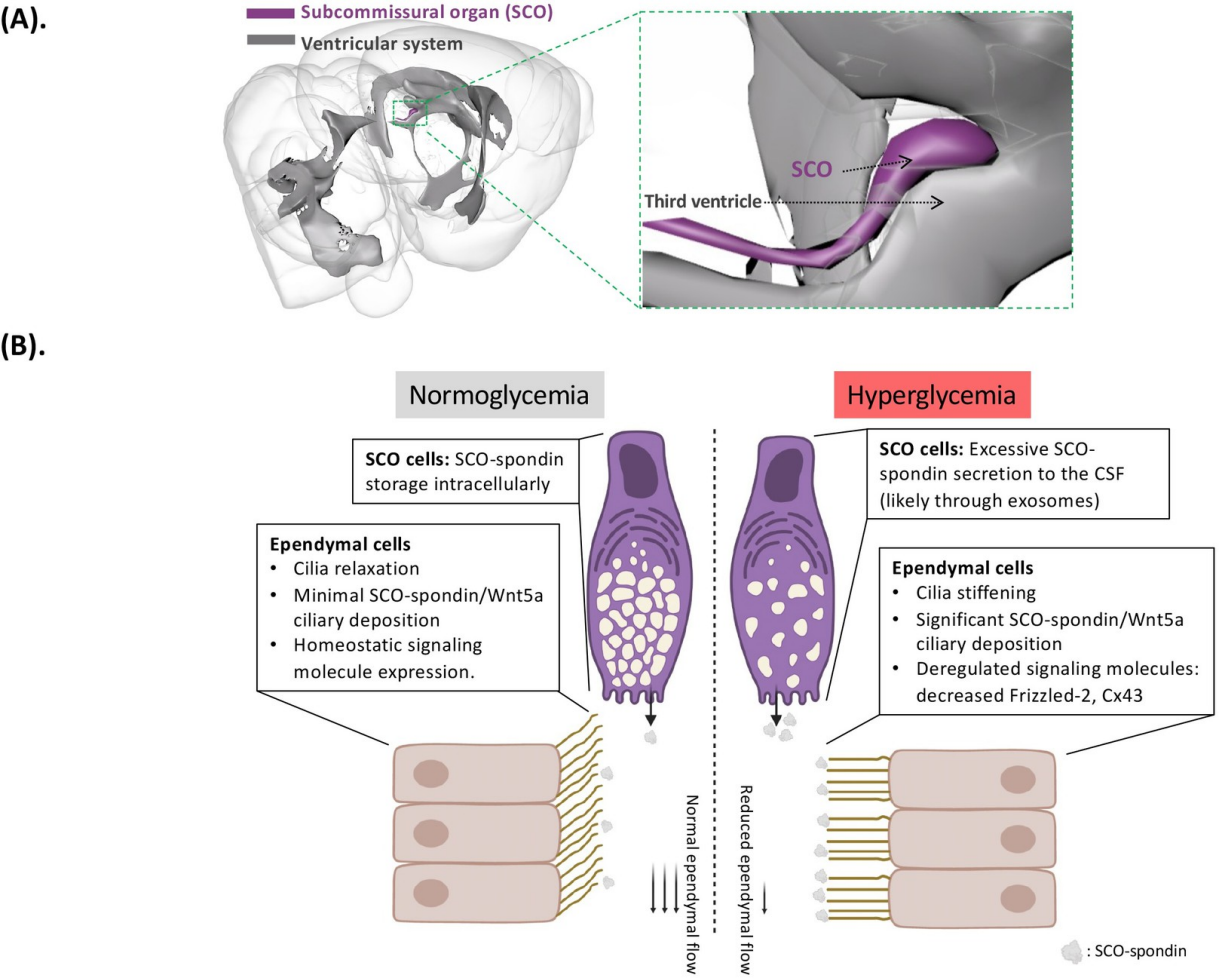
Hyperglycemia induces SCO secretion of SCO-spondin to decrease ependymal flow in the third ventricle. (**A**) Spatial proximity of SCO and the third ventricle. SCO is located immediately on the roof of the third ventricle. Secretory compartments on the apical aspect of SCO cells directly contact the CSF, allowing SCO-secreted molecules to readily communicate with ependyma. This illustration is created with the 3D Brain Composer using The Scalable Brain Atlas published by Rembrandt Bakker, Paul Tiesinga, and Rolf Kötter in 2015 (The Scalable Brain Atlas: instant web-based access to public brain atlases and related content. DOI: 10.1007/s12021-014-9258-x). (**B**). In this issue, Nualart and colleagues show that increased CSF glucose reduces ependymal flow by modifying ependymal cilia function. Under normoglycemia (left), SCO-spondin is largely stored in SCO cells, with only minimal SCO-spondin detected extracellularly. Accordingly, ependyma establish a normal CSF flow along the ventricular surface. Motile cilia of ependymal cells are properly aligned and angled, suggesting a relaxed and flexible state for ciliary beating. However, under hyperglycemia (right), increased CSF glucose induces SCO-spondin secretion to the CSF and substantially reduces ependymal flow. With SCO-spondin proteins deposited on the ependyma, motile cilia of ependymal cells appear stiffened and perpendicular to the ventricular surface. In addition, hyperglycemia disrupts critical signaling molecules in ependymal cells, such as Frizzled-2 and Cx-43. This diagram is created with BioRender.com.

Glucose provides energy for the body and is the primary fuel source for neuronal metabolism. The brain senses glucose changes: for example, increased blood glucose concentration after a big meal leads to satiety. Cerebrospinal fluid (CSF) glucose fluctuates with blood glucose, with CSF glucose approximately 60% of plasma levels [1]. The hypothalamus is primarily implicated in brain glucose sensing: subpopulations of hypothalamic neurons either directly sense glucose changes or indirectly receive glucose-related information from specific glial cells like tanycytes that contact CSF [2]. In this issue, Nualart and colleagues [3] further probe glucose sensing in the brain, describing how the subcommissural organ (SCO) responds to increased CSF glucose by secreting signaling molecules into the CSF-filled third ventricle, thereby decreasing local ependymal CSF flow (**Fig 1**).

Nualart and colleagues focus on the SCO because of this glandular organ’s ability to take up glucose (supported by a uniquely high expression of GLUT2, a critical glucose transporter) and secrete signaling molecules (such as SCO-spondin and Wnt5a). Given the anatomical proximity of the SCO to CSF in the third ventricle (**Fig 1A**), the authors investigate how the SCO could influence ependymal cells that line the ventricle to modulate their motile cilia, which actively stroke in unison to facilitate local CSF movement. To do this, they compare basal and hyperglycemic conditions in rodent models. They find that in contrast to normoglycemia, hyperglycemia facilitates release of SCO-enriched spondin protein (SCO-spondin) into CSF, which has an “immobilizing” effect on ependymal cilia. This observation is supported both by electron microscopic analyses of ependymal cilia and ex vivo ependymal flow analyses. In scanning electron micrographs, ependymal cilia under normoglycemic basal conditions appear “soft and relaxed” and are properly angled and aligned, suggesting strong ciliary beating. In contrast, ependymal cilia under hyperglycemic conditions show evidence of increased stiffness, appearing clustered and perpendicular to the ependymal wall in scanning micrographs. Furthermore, ependymal flow in the third ventricle is significantly inhibited by hyperglycemia, demonstrated by quantifying real-time speed of fluorescent microbeads moving along ependymal surfaces ex vivo. Closer examination of ex vivo ependyma through high-resolution confocal microscopy reveals that microbeads, which are only minimally detected on normal ependyma, seem to be trapped within ependymal cilia upon glucose challenge. These outcomes of altered ependymal function, structure, and signaling are at least partially mediated by the secretion of SCO-spondin and Wnt5a proteins into the third ventricle by the SCO (**Fig 1B**).

What are the potential outcomes of ependymal flow slowdown? Slower flow could extend the duration of glucose detection, since it would prolong the exposure time window for glucose-sensing organs to sample CSF glucose. However, chronic decreased ependymal flow is also strongly associated with hydrocephalus, a neurological disorder characterized by excessive buildup of CSF in brain ventricles [4]. Indeed, CSF composition and flow (i.e., speed and directionality) contribute to volume homeostasis [5,6]. Nualart and colleagues provide compelling evidence demonstrating reduced ependymal flow and altered local CSF composition caused by hyperglycemia-induced SCO-spondin secretion. Notably, these observations are all made in the third ventricle, which connects the lateral ventricles to the fourth ventricle through the only 2 existing physiological narrowings of the brain ventricular system: the foramen of Monro and the aqueduct. Slowdown of flow through these narrowest portions of the ventricular system could disproportionately disrupt bulk CSF movement. When analyzing adult human brain, in which the SCO has diminished from its existence earlier in development, the authors discover certain SCO elements, including spondin expression, in neighboring third ventricle ependyma. Due to sequence similarity, the authors posit that the human ependymal spondin they detect is not SCO-spondin, but could be the closely related human R-spondin 4. The mRNA for R-spondin 4 is detected in human pediatric ependymal cells, further supporting the hypothesis that it could account for the spondin signals observed in adult human brains. This finding suggests that roles specific to the SCO in other species, including CSF-glucose sensing, may still be relevant in humans if carried out by this subpopulation of ependymal cells. Since hyperglycemia is a leading symptom of diabetes (both type 1 and type 2), these data in humans could further inform a mechanism for the clear, reproducible observation that diabetes drives idiopathic normal pressure hydrocephalus [7]. According to the United States Centers for Disease Control and Prevention, 37.3 million Americans are living with diabetes, and 96.0 million American adults have prediabetes, an intermediate hyperglycemic borderline condition highly likely to progress to diabetes within 5 years [8]. These findings sound the alarm: uncontrolled glucose levels could eventually impair CSF homeostasis.

Results from this study motivate some immediate follow-up investigations to further expand and contextualize the main claims. First, applying available in vivo CSF analysis methods, such as tracer dye- or particle tracing-based intravital imaging techniques [9], will supplement the authors’ ex vivo analysis and provide more holistic context for CSF flow and dynamics in hyperglycemia. Second, further investigations of the mechanisms identified in the current study by modeling chronic hyperglycemia will inform similarities between reduced ependymal flow induced by a single, acute intraperitoneal glucose dose and pathological chronic hyperglycemia. Finally, additional studies investigating downstream SCO-spondin effects, including Wnt5a/Frizzled-2/Cx-43 will bolster the mechanism proposed here. Thus, this study immediately inspires next mechanistic steps in vivo and in chronic hyperglycemia.

More broadly, Nualart and colleagues open novel lines of investigation, motivating researchers to join this exciting field. One topic inspired by this work that is ripe for future study is whether the SCO contributes to glucose sensing in the hypothalamus. In particular, can the SCO regulate activity of tanycytes (the specialized ependymal cells lining the third ventricle with a uniquely high glucose-sensing capability) that establish connections with hypothalamic neurons? In addition, what does the SCO secretome contain beyond spondin and Wnt5a? Since the SCO is a glandular secretory tissue that releases substances directly into the CSF, a systemic understanding of its secretome may further reveal its regulatory role in brain health. Of note, GLUT2 expression in the SCO was lost in aged brains [3], but whether this loss leads to disrupted SCO glucose sensing remains to be determined. Moreover, understanding when this signaling axis emerges in development could illuminate links between gestational diabetes and pediatric hydrocephalus. Finally, since SCO secretion into the CSF regulates ependymal cilia, does the SCO also modulate choroid plexus epithelial cell cilia? The pioneering work by the authors in this study implicates CSF as a medium for crucial crosstalk during glucose sensing and sparks exciting avenues for future investigations.
